# Assessment of the sensitivity of ^2^H MR spectroscopy measurements of [2,3‐^2^H_2_]fumarate metabolism for detecting tumor cell death

**DOI:** 10.1002/nbm.4965

**Published:** 2023-05-22

**Authors:** Friederike Hesse, Alan Wright, Flaviu Bulat, Felix Kreis, Kevin M. Brindle

**Affiliations:** ^1^ Cancer Research UK Cambridge Institute Cambridge UK; ^2^ Department of Radiology University of Cambridge Cambridge UK; ^3^ Guy's and St Thomas's NHS Foundation Trust St Thomas' Hospital London UK; ^4^ Department of Chemistry University of Cambridge Cambridge UK; ^5^ Department of Biochemistry University of Cambridge Cambridge UK

**Keywords:** deuterium, fumarate, necrosis, tumor

## Abstract

Imaging the metabolism of [2,3‐^2^H_2_]fumarate to produce malate can be used to detect tumor cell death post‐treatment. Here, we assess the sensitivity of the technique for detecting cell death by lowering the concentration of injected [2,3‐^2^H_2_]fumarate and by varying the extent of tumor cell death through changes in drug concentration. Mice were implanted subcutaneously with human triple negative breast cancer cells (MDA‐MB‐231) and injected with 0.1, 0.3, and 0.5 g/kg [2,3‐^2^H_2_]fumarate before and after treatment with a multivalent TRAlL‐R2 agonist (MEDI3039) at 0.1, 0.4, and 0.8 mg/kg. Tumor conversion of [2,3‐^2^H_2_]fumarate to [2,3‐^2^H_2_]malate was assessed from a series of 13 spatially localized ^2^H MR spectra acquired over 65 min using a pulse‐acquire sequence with a 2‐ms BIR4 adiabatic excitation pulse. Tumors were then excised and stained for histopathological markers of cell death: cleaved caspase 3 (CC3) and DNA damage (terminal deoxynucleotidyl transferase dUTP nick end labeling [TUNEL]). The rate of malate production and the malate/fumarate ratio plateaued at tumor fumarate concentrations of 2 mM, which were obtained with injected [2,3‐^2^H_2_]fumarate concentrations of 0.3 g/kg and above. Tumor malate concentration and the malate/fumarate ratio increased linearly with the extent of cell death determined histologically. At an injected [2,3‐^2^H_2_]fumarate concentration of 0.3 g/kg, 20% CC3 staining corresponded to a malate concentration of 0.62 mM and a malate/fumarate ratio of 0.21. Extrapolation indicated that there would be no detectable malate at 0% CC3 staining. The use of low and nontoxic fumarate concentrations and the production of [2,3‐^2^H_2_]malate at concentrations that are within the range that can be detected clinically suggest this technique could translate to the clinic.

## Graphical abstract

Deuterium imaging of the production of labeled malate from injected [2,3‐^2^H_2_]fumarate can be used to detect tumor cell death post‐treatment. Here, we have determined the optimal fumarate concentration for cell death detection and demonstrated that the rate of malate production is linearly related to the degree of cell death determined histologically.

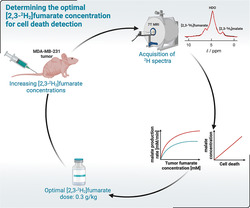

**Abbreviations**
CC3: cleaved caspase 3
DMEM: Dulbecco's Modified Eagle Medium
FOV: field of view
FSE: fast spin echo
STR: Short Tandem Repeats
TNF: tumor necrosis factor
TRAIL: TNF‐related apoptosis‐inducing ligand
TUNEL: terminal deoxynucleotidyl transferase dUTP nick end labeling

## 1 INTRODUCTION

Imaging cell death can give an early indication of tumor treatment response and the effectiveness of therapy, where the degree of tumor cell death can be an indicator of treatment outcome.^1^, ^2^ Currently, assessment of tumor treatment response in the clinic focuses primarily on changes in tumor size.^3^, ^4^ However, these may take weeks to appear following treatment, reducing the therapeutic window and impacting patient survival. Despite the introduction of several promising cell death imaging agents in preclinical studies, to date, clinical trials of these agents have failed to produce robust results.^5^

We have shown previously that tumor cell death post‐treatment can be detected in mouse tumor models in vivo through the production of hyperpolarized ^13^C‐labeled malate in animals injected intravenously with hyperpolarized [1,4‐^13^C_2_]fumarate.^6^, ^7^ Fumarate is hydrated in the reaction catalyzed by the enzyme fumarase to produce malate. In necrotic cells, loss of plasma membrane integrity results in fumarate rapidly gaining access to the enzyme and an increased rate of malate production. More recently, we have shown, in EL4 murine lymphomas, in human colorectal (Colo205) and breast cancer (MDA‐MB‐231) xenografts,^8^ and in orthotopically implanted patient‐derived glioblastoma xenografts,^9^ that ^2^H MRS and MRSI measurements can be used similarly to detect cell death post‐treatment by measuring the increase in ^2^H‐labeled malate concentration following intravenous [2,3‐^2^H_2_]fumarate injection. Although the sensitivity of label detection is less for the ^2^H experiment, the contrast, expressed as the labeled malate/fumarate ratio, is greater, as the labeled malate concentration can build up over a much longer period of time. The short half‐life of the hyperpolarized ^13^C label limits the degree of contrast that can be achieved in the ^13^C MR experiment.^9^ Here, we have investigated the sensitivity of the ^2^H experiment for detecting tumor cell death post‐treatment in MDA‐MB‐231 xenografts by both varying the injected [2,3‐^2^H_2_]fumarate concentration and by varying the extent of tumor cell death through changes in drug dose.

## 2 METHODS

### 2.1 Cell culture

Human triple negative breast cancer MDA‐MB‐231 cells were cultured in Dulbecco's Modified Eagle Medium (DMEM) (Gibco, UK) containing 10% FBS. When confluent, the cells were washed with PBS (Gibco) and dissociated with 0.25% trypsin (Gibco). To minimize phenotypic drift, cells were implanted after two passages from thawing. A Vi‐Cell counter (Vi‐Cell XR, Beckman Coulter) was used to assess viability and cell count. Cells tested negative for mycoplasma, and were genotyped using Short Tandem Repeats (STR), yielding a 100% match to cells in the Cellosaurus STR database (MDA‐MB‐231).

### 2.2 Tumor implantation and treatment

MDA‐MB‐231 cells were resuspended in a 1:1 mixture of Matrigel (Corning) and complete DMEM and implanted subcutaneously on the upper back and between the fore limbs of 10–12‐week‐old female BALB/c Nu/Nu mice (Charles River) at 7 x 10^6^ cells. ^2^H spectroscopy measurements were conducted when the tumors reached ~ 1 cm^3^ (~35 days following implantation). The animals were treated with an escalating dose of a tumor necrosis factor (TNF)‐related apoptosis‐inducing ligand (TRAIL) receptor 2 agonist (MEDI3039),^10^ with 0.1, 0.4, and 0.8 mg/kg being administered intravenously 24 h before imaging. Animal experiments were carried out in compliance with project and personal licenses issued under the Animals (Scientific Procedures) Act of 1986 by the Home Office, UK, and were approved by the Cancer Research UK Cambridge Institute Animal Welfare and Ethical Review Body.

### 2.3 MR spectroscopy in vivo

Animals were anesthetized by inhalation of 2% isoflurane in air/O_2_ (75%/25%, 2 L/min). Breathing rate and body temperature were monitored and body temperature was maintained with a stream of warm air. ^2^H spectroscopy was performed at 7 T (Agilent, Palo Alto, CA, USA) using a home‐built 10‐mm diameter single‐loop surface coil placed over the tumor, as described previously.^8^, ^9^, ^11^ Tumors were localized in axial ^1^H images acquired using a T_2_‐weighted fast spin echo (FSE) pulse sequence (repetition time [TR], 2 s; echo time [TE], 50 ms; field of view [FOV], 36 x 36 mm, 256 x 256 matrix; slice thickness, 2 mm; 10 slices). Disodium [2,3‐^2^H_2_]fumarate (Cambridge Isotope Laboratories) was dissolved in water and 0.2 mL was administered intravenously via a tail vein catheter to give concentrations of 0.1, 0.3, and 0.5 g/kg body weight. Infusion started 5 min after the start of spectral acquisition and lasted for a period of 20 min. Thirteen serial 5‐min ^2^H spectra were acquired with a 2‐ms BIR4 pulse,^12^ with a nominal flip angle of 67°, a TR of 140 ms and 2142 signal averages^8^ over a time period of 65 min. Localization of signal to the tumors was achieved by the excitation profile of the surface coil. Spectra were zero‐ and first‐order phase‐corrected, and the peaks modeled with the AMARES algorithm implemented in the OXSA toolbox,^13^, ^14^ which uses a time‐domain fitting algorithm and employs prior input knowledge to optimize the accuracy of fit.^13^ The peak integrals were determined from the time domain modeling. The area under the peak was determined from the integral of the fitted line shape. The intensity of the HDO peak at the first time point was assumed to correspond to the natural abundance of deuterium in tumor water. The signals were corrected for saturation and for the number of ^2^H nuclei in [2,3‐^2^H_2_]fumarate, as described previously.^8^ The concentration of malate was estimated from the intensity of the upfield resonance, assuming that it has a similar T_1_ to the fumarate resonance.

The natural abundance of deuterium in tumor water was estimated from the measured concentration of ^2^H in tap water and by assuming a tissue water content fraction of 0.8.^15^ The natural abundance ^2^H content of Cambridge tap water was measured by adding 5 mM formate‐d, as a reference standard, to 1 mL of water, and acquiring fully relaxed ^2^H spectra at 310 K using the ^2^H coil of a 5‐mm ^1^H/broadband inverse detection probe in a 14.1‐T NMR spectrometer (Bruker Spectrospin). Spectra were acquired using a 90° pulse, a TR of 10 s with a 2000 Hz spectral width, into 1024 datapoints, and were the sum of 1024 transients. Spectra were phased, baseline corrected, and the peak integrals were calculated using Topspin (Bruker Spectrospin). The amplitude of the water resonance was normalized to the formate‐d peak to give an absolute ^2^H concentration of 15.61 mM. The tumor HDO peak, when corrected for tissue water fraction, was therefore assumed to correspond to 12.48 mM ^2^H.

### 2.4 Histology and immunohistochemistry

Tumors were excised immediately following the imaging session and transferred into 10% formalin for 24 h, followed by 70% ethanol, and embedded in paraffin before sectioning into two 10‐μm thick sections spaced 100 μm apart. Sections were stained with hematoxylin and eosin (ST020 Multistainer—Leica Microsystems) and with a rabbit monoclonal anti‐CC3 antibody (Cell Signaling Technology) at a 1:400 dilution, and a donkey antirabbit secondary biotinylated antibody (Jackson Immuno‐Research Laboratories) using Leica's Polymer Refine Kit (Leica Microsystems) on an automated Bond platform (Leica Biosystems). Sections were also stained using a terminal deoxynucleotidyl transferase dUTP nick end labeling (TUNEL) colorimetric system kit (Promega Benelux BV). Slides were scanned at 20 × magnification with a resolution of 0.5 μm per pixel on an Aperio AT2 (Leica Biosystems). The whole of the two tumor sections were analyzed using a CytoNuclear v. 1.6 algorithm on HALO v. 3.0.311.293 (Indica Labs) to quantify the percentage of positive cells.

### 2.5 Statistical analysis

Statistical and graphical analyses were performed using Prism v. 9.0 (GraphPad). Analysis of variance (ANOVA) was used followed by Tukey's post hoc test for multiple comparisons of groups to determine significance. Pearson’s R test was used to assess the significance of the correlation analysis. *p* values are summarized in the figures as: *p = 0.01–0.05; **p = 0.001–0.01; ***p = 0.0001–0.001; and ****p < 0.0001. Data are shown as mean ± SD, unless stated otherwise.

## 3 RESULTS

**FIGURE 1:**
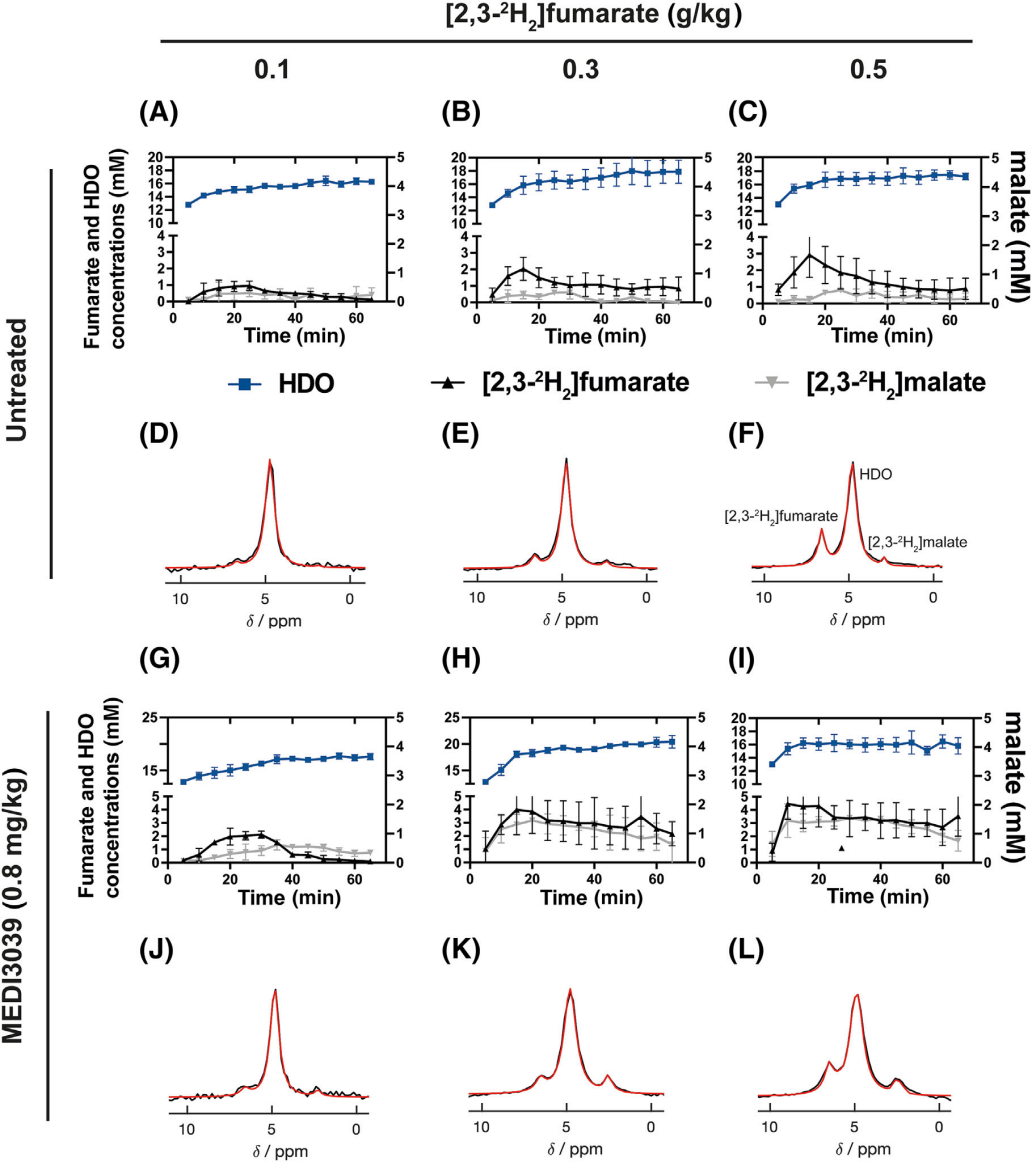
^2^H MR spectroscopic measurements of labeled fumarate, malate and water concentrations in MDA‐MB‐231 tumors following injection of increasing concentrations of [2,3‐^2^H_2_]fumarate (A–C,G–I) before (A–C) (*n* = 3) and 24 h after treatment with 0.8 mg/kg MEDI3039 (G–I) (*n* = 3). The corresponding representative spectra, the sum of 12 spectra recorded over 60 min, are shown in (D–F) and (J–L). [2,3‐^2^H_2_]fumarate was infused at three different concentrations, ranging from 0.1 (*n* = 3) (A,D,G,J), 0.3 (*n* = 3) (B,E,H,K) and 0.5 g/kg (*n* = 3) (C,F,I,L), and started 5 min following the start of acquisition of the first spectrum.

Deuterium‐labeled fumarate, malate and water concentrations were measured in subcutaneous MDA‐MB‐231 breast cancer xenografts following intravenous injection of [2,3‐^2^H_2_]fumarate at 0.1, 0.3, and 0.5 g/kg and following treatment with 0.1, 0.4, and 0.8 mg/kg MEDI3039. The data for untreated tumors and tumors treated with 0.8 mg/kg MEDI3039 are shown in Figure 1, and the rest of the data are shown in Figure S1 in the supporting information. Individual 5‐, 10‐, and 20‐min spectra acquired starting at 20 min following injection of 0.3 g/kg fumarate and 24 h after treatment with 0.8 mg/kg MEDI3039 are shown in Figure S2. The fumarate concentrations in the tumors at 20 min postinjection, when the concentration started to plateau, are shown in Table 1. There were significant increases in tumor fumarate concentrations with increases in injected dose and following treatment. The increase following treatment reflects an increase in tumor perfusion, as has previously been observed in this tumor model.^8^ At higher injected fumarate concentrations, malate build‐up was detectable at earlier time points, which was most evident in tumors treated with 0.8 mg/kg MEDI3039. The malate concentration increased significantly with increases in the concentration of injected [2,3‐^2^H_2_]fumarate (Figure S3) at drug concentrations of 0.4 mg/kg or higher. The malate/fumarate ratio also increased relative to untreated controls at all drug and fumarate concentrations. These increases were significant at 0.4 and 0.8 mg/kg MEDI3039 (Figure S4), although not at 0.1 mg/kg MEDI3039 (0.1 g/kg fumarate, *p* = 0.08; 0.3 g/kg fumarate, *p* = 0.28; 0.5 g/kg fumarate, *p* = 0.25). The coefficients of variance for the fumarate and malate signals are shown in Table S1 and Figure S5. The coefficients of variance ranged from 8% to 21% for fumarate and from 6% to 35% for malate, with the values decreasing with increasing tumor metabolite concentrations.

**TABLE 1:** Dependence of tumor fumarate concentrations on the injected fumarate dose and on drug treatment.

Injected [2,3‐^2^H_2_]fumarate concentration (g/kg)				
	0.1	0.3	0.5	1
Drug dose mg/kg	[2,3‐^2^H_2_]fumarate concentration in the tumor (mM)			
Untreated	0.89 ± 0.15	1.45 ± 0.51	2.18 ± 0.91	3.03 ± 1.10
0.1	1.02 ± 0.18	2.08 ± 0.73	2.43 ± 0.49	
0.4	1.01 ± 0.24	2.01 ± 0.31	2.95 ± 0.36	
0.8	1.93 ± 0.71	3.81 ± 1.07	4.16 ± 1.19	4.91 ± 1.27

The tumor fumarate concentrations were determined from localized ^2^H spectra acquired at 20 min after the start of [2,3‐^2^H_2_]fumarate infusion at the indicated concentrations. Animals were treated with MEDI3039 by intravenous injection of the drug, at the indicated concentrations, 24 h before imaging.

**FIGURE 2:**
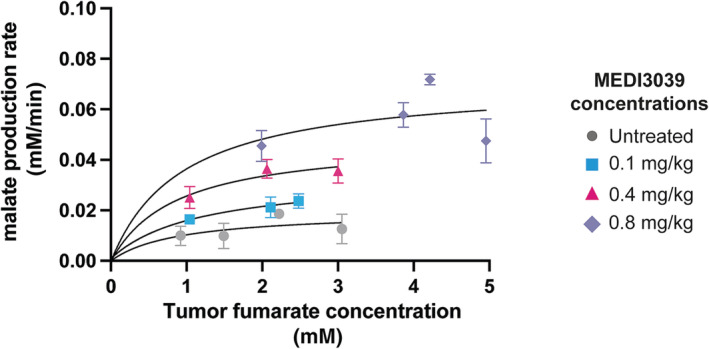
Apparent Km of fumarase for tumor fumarate. Apparent malate production rates were calculated between 0 and 20 min following fumarate administration and plotted against the tumor fumarate concentration at 20 min. The curves were fit to the Michaelis–Menten equation to obtain an estimate of Km and Vmax. The three datapoints at tumor fumarate concentrations between 4 and 5 mM were obtained following injection of 1 g/kg [2,3‐^2^H_2_]fumarate and were taken from reference.^8^

**FIGURE 3:**
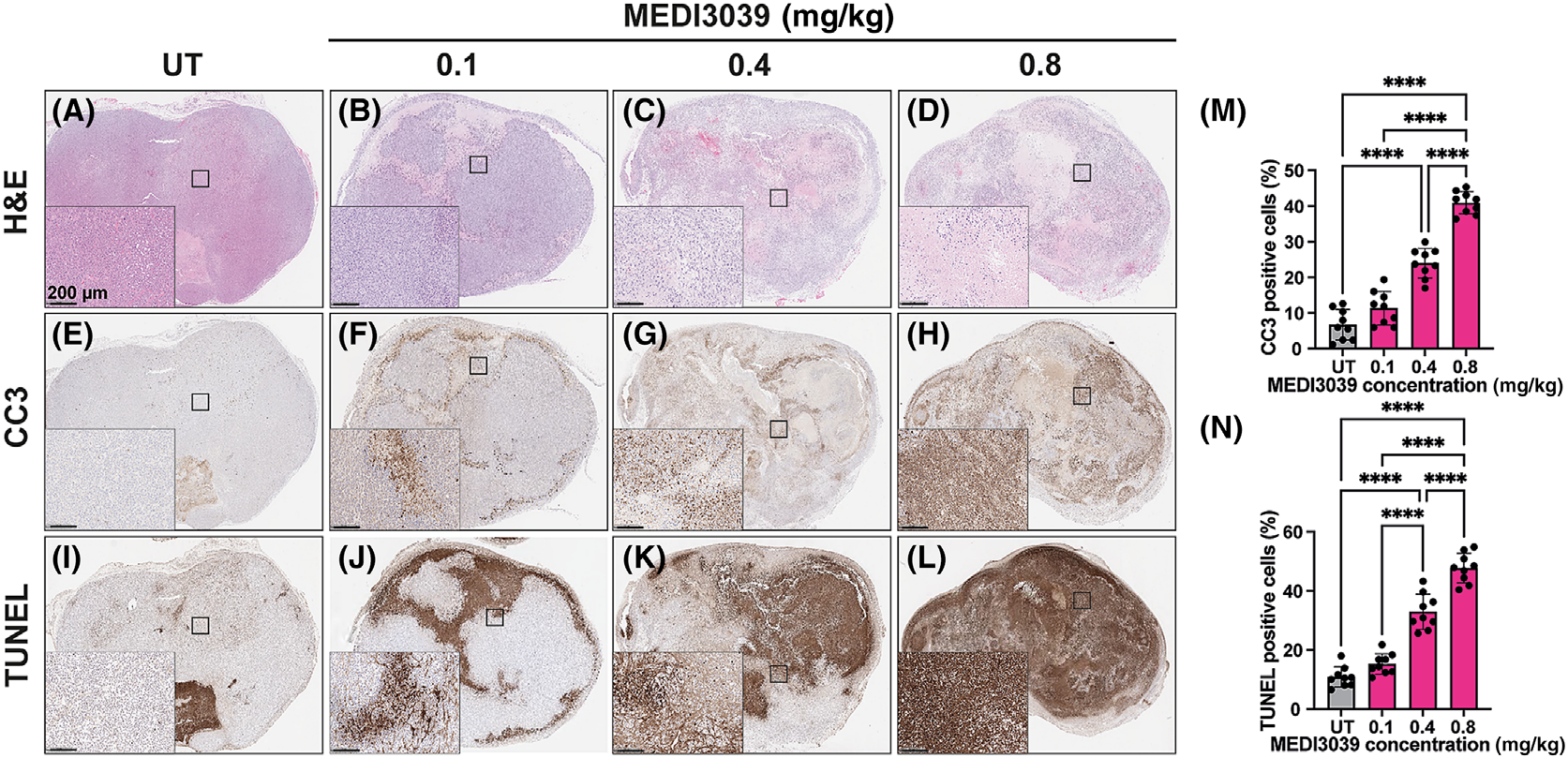
Histological assessment of tumor cell death before and at 24 h after treatment with increasing MEDI3039 doses (0.1, 0.4, and 0.8 mg/kg). Representative tumor sections stained with (A–D) H&E, (E–H) CC3, and (I–L) TUNEL. The percentage of (M) CC3 and (N) TUNEL positive cells in untreated tumors and after treatment with increasing doses of MEDI3039. Data are shown as mean ± SD, *n* = 9 per group; ****p < 0.0001. CC3, cleaved caspase 3; H&E, hematoxylin and eosin; TUNEL, terminal deoxynucleotidyl transferase dUTP nick end labeling; UT, untreated.

The initial rate of malate production was estimated from the malate concentration at 20 min and plotted against the tumor fumarate concentration at this time point (Figure 2). Fitting of these data to the Michaelis–Menten equation (Equation 1) gave the values for the apparent Vmax and Km shown in Table 2.

(1) $$v=\frac{\text{Vmax}\left[S\right]}{\mathrm{Km}+\left[S\right]}$$

where v is the initial rate of malate production (mM/min), [S] is the tumor fumarate concentration (mM), Km is the Km of fumarase for fumarate (mM), and Vmax (mM/min) is the maximal velocity of the enzyme at saturating fumarate concentrations. There was no increase in malate production rate at tumor fumarate concentrations greater than 2 mM, which in this tumor model was obtained with an injected fumarate concentration of 0.3 g/kg and above, and therefore the enzyme appears to be saturated at this fumarate concentration (Figure 2). The apparent Vmax increased with increasing drug concentrations, reflecting the increased tumor cell death at the higher drug concentrations (Figure 3), and therefore the increased access of the injected fumarate to intracellular fumarase.

**TABLE 2:** Apparent Km and Vmax values for tumor fumarase.

Drug dose mg/kg	Km (mM)	Vmax (mM/min)
Untreated	0.98	0.02
0.1	1.18	0.03
0.4	0.88	0.05
0.8	0.95	0.07

*Note*: The apparent Km of tumor fumarase for fumarate and the Vmax at different injected drug concentrations were determined by fitting the data shown in Figure 2 to the Michaelis–Menten equation.

**FIGURE 4:**
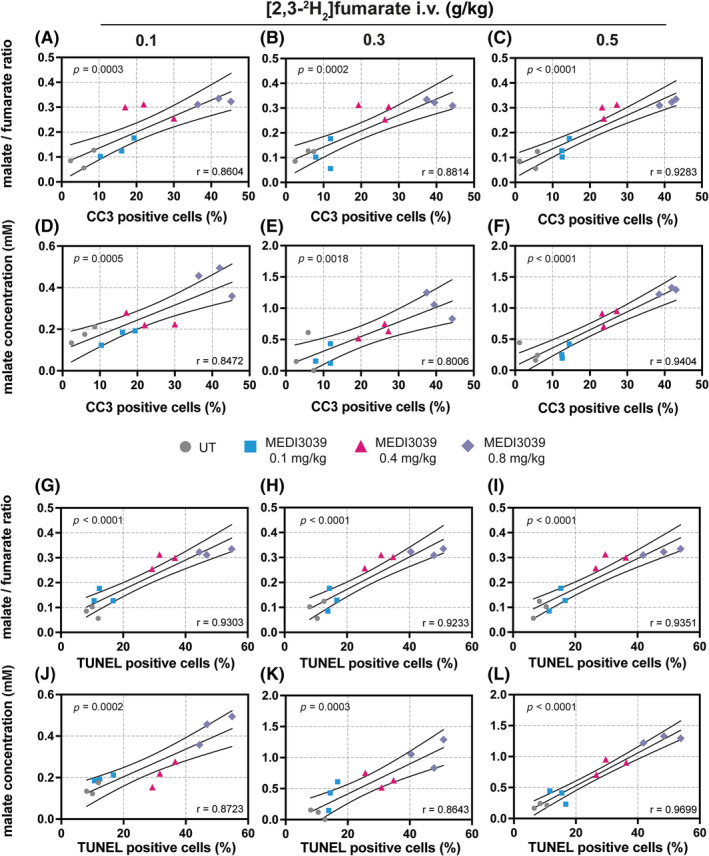
Pearson correlation analysis of histological markers of cell death, (A–F) CC3 and (G–L) TUNEL % positive cells, with the malate/fumarate ratio and malate concentration (mM) obtained by summing the spectra between 20 and 60 min after fumarate injection. Tumor‐bearing mice were treated with MEDI3039 at 0.1, 0.4, or 0.8 mg/kg and 24 h later injected with 0.1, 0.3, and 0.5 g/kg [2,3‐^2^H_2_]fumarate. Following imaging, the tumors were excised, sectioned, and stained for CC3 and TUNEL. Two‐tailed, Pearson *p* and *r* values are shown. CC3, cleaved caspase 3; i.v., intravenous; TUNEL, terminal deoxynucleotidyl transferase dUTP nick end labeling; UT, untreated.

The extent of drug‐induced cell death was determined by histological assessment of cells positive for CC3 and TUNEL (Figure 3). Increasing the drug dose resulted in parallel increases in CC3 (Figure 3E–H,M) and TUNEL (Figure 3I–L,N) staining. The malate/fumarate ratios and malate concentrations were linearly correlated with CC3 (Figure 4A–F) and TUNEL staining (Figure 4G–L) at all three injected fumarate concentrations, but both were higher at 0.3 and 0.5 g/kg.

## 4 DISCUSSION

In a previous study with EL4 murine lymphomas and human colorectal (Colo205) xenografts, and with the breast cancer (MDA‐MB‐231) xenografts used in this study, we showed that following treatment there was a 10‐ to 18‐fold increase in the tumor malate/fumarate signal ratio following intravenous injection of [2,3‐^2^H_2_]fumarate at 1 g/kg.^8^ Here, we have assessed the sensitivity of this experiment for detecting cell death by decreasing the injected [2,3‐^2^H_2_]fumarate concentration and by varying the amount of cell death through changes in drug dose in the MDA‐MB‐231 breast cancer xenografts.

Fitting the rate of labeled malate production at different intratumoral [2,3‐^2^H_2_]fumarate concentrations to the Michaelis–Menten equation allowed estimation of an apparent Vmax and Km of tumor fumarase for fumarate, which gave values for the Km of between 0.88 and 1.18 mM. An early determination of the fumarase Km for fumarate gave a value of 5 μM,^16^ however, a more recent estimate gave a value of 0.4 mM for the human enzyme at pH 6.5.^17^ Although, in estimating an apparent Km, we have neglected the effects of changes in tumor fumarate concentration during the first 20 min after the start of fumarate injection and loss of labeled malate due to washout and exchange of the deuterium label with solvent,^8^ our data are more consistent with the higher Km of 0.4 mM.

Plots of malate/fumarate ratio and malate concentration versus the percentage of dead cells (CC3 and TUNEL positive) were linear with intercepts of near zero, demonstrating that there is little or no malate production in viable tumor tissue and that there is little wash in of malate from other tissues, as was demonstrated previously using blood sampling.^8^ These plots allow estimation of the degree of contrast that will be generated, and therefore the sensitivity for detecting cell death, at any level of cell death for a given injected fumarate concentration. At an injected fumarate dose of 0.3 g/kg, 20% CC3 staining corresponded to a tumor malate concentration of 0.62 mM and a malate/fumarate ratio of 0.21. In principle, these plots allow estimation of the degree of cell death‐dependent tumor contrast that would be generated for any tumor if it is assumed that intracellular fumarase activity and the degree of exposure of injected fumarate to the enzyme during necrotic cell death is similar between the breast cancer tumor model used here and other tumor types. In this regard, it is notable that the activities of the enzyme in EL4 murine lymphomas, human colorectal (Colo205) xenografts and breast cancer (MDA‐MB‐231) xenografts are similar and all three tumors showed similar cell death‐dependent increases in the malate/fumarate ratio post treatment.^8^ In orthotopically implanted patient‐derived xenograft models of glioblastoma treated with chemoradiation, we observed even greater contrast with 20% CC3 staining, corresponding to a malate concentration of 1.38 and a malate/fumarate ratio of 0.71.^9^ In a study in breast cancer patients treated with chemotherapy, biopsy sampling showed a median increase of 3.4% of TUNEL positive cells in responders compared with −0.1% in nonresponders.^18^ However, there was a marked variation in TUNEL staining, which may reflect inadequate sampling of the tumor mass by biopsy, with some responding patients showing large increases in staining, from 0.6% to 6.2%, 8.8% to 15%, 1.7% to 8.2%, and 0.4% to 1.6%. Similar results were obtained in a more recent study in patients treated with trastuzumab, where there was a median increase in CC3 staining, from 3.5% to 4.7%, with again a wide variation in the increases between patients, with some showing an apoptotic index of greater than 10% at 1 week after treatment.^19^ Assuming cell death doubles from 5% to 10% post‐treatment, then based on the data presented here for MDA‐MB‐231 xenografts, injection of 0.3 g/kg fumarate would give a 40% increase in the malate/fumarate ratio. Moreover, if the image voxel encompasses the entire volume of the tumor then the experiment effectively integrates all the cell death that is present and thus avoids the sampling error that plagues biopsy.

An advantage of the technique is that it probably only detects necrotic cells that have been killed recently by treatment because fumarase is likely to leak out of treated tumors over time. Cells that were killed by previous treatments are less likely to be detected. A potential drawback of the technique is that it is less likely to detect cell death if the number of necrotic cells increases slowly or if the timing of the increase in cell death post‐treatment is unknown. The treatments that we have used here and in previous studies with hyperpolarized ^13^C‐labeled^6^, ^7^ and ^2^H‐labeled fumarate^8^, ^9^, ^11^ led to relatively rapid increases in tumor cell death.

The method should be translatable to the clinic because it can generate malate concentrations in excess of 1 mM, which, based on the concentrations of glucose, lactate, and glutamate/glutamine (Glx) measured in clinical studies,^20^, ^21^ should be sufficient for detection on clinical 3‐T MR scanners. For example, Glx and lactate concentrations of approximately 1–3 mM were detectable at 3 T in 10‐min spectra acquired from an isotropic 3.2‐cm voxel in the brains of normal volunteers given [6,6′‐^2^H_2_]glucose orally (0.75 g/kg).^21^ Even the lowest concentration of [2,3‐^2^H_2_]fumarate (0.1 g/kg) used here generated a detectable malate signal ~35 min after administration, which suggests that by adjusting the time of data acquisition, detection of malate produced from even lower concentrations of fumarate may be possible. Moreover, as discussed previously,^8^ the chemical shift separations of the fumarate (6.5 ppm), water (4.7 ppm), and malate (2.4 ppm) resonances are greater than those for the water, glucose (3.9 ppm), Glx (2.4 ppm), and lactate (1.35 ppm) resonances, and resolved resonances from Glx and lactate and partially resolved resonances from water and glucose have been observed at 3 T.^21^ Clinical translation would be facilitated by oral administration and we have shown recently in EL4 lymphomas that oral administration of 2 g/kg [2,3‐^2^H_2_]fumarate produces similar malate/fumarate ratios to those resulting from intravenous injection of 1 g/kg [2,3‐^2^H_2_]fumarate.^11^ If we assume that, in general, an oral dose must be twice that of an injected dose, then for a 60‐kg patient an injected dose of 0.3 g/kg would be equivalent to an oral dose of 36 g [2,3‐^2^H_2_]fumarate. Fumarate has been given orally to humans in doses ranging from 5 to 30 g to study its potential laxative effects. Up to six doses were given with no evidence of renal or liver impairment,^22^ demonstrating the feasibility of using oral [2,3‐^2^H_2_]fumarate and ^2^H MRSI to image tumor cell death in the clinic.

## 5 CONCLUSION

We showed previously that tumor cell death could be detected from the labeled malate produced following intravenous injection of 1 g/kg [2,3‐^2^H_2_]fumarate. We have shown here that cell death can still be detected with injected [2,3‐^2^H_2_]fumarate concentrations as low as 0.1 g/kg, although 0.3 g/kg was optimal, and that the malate/fumarate ratio was linearly dependent on the extent of tumor cell death.

## Supporting information


**Table S1** Coefficients of variance for fumarate
**Figure S1.** ^2^H MR spectroscopic measurements of labeled fumarate, malate and water concentrations in MDA‐MB‐231 tumors following injection of increasing concentrations of [2,3‐^2^H_2_]fumarate (**A‐C, G‐I**) 24 h after treatment with 0.1 mg/kg (**A‐C**) (n=3) and 0.4 mg/kg (**G‐I**) (n=3) MEDI3039. The corresponding representative spectra, the sum of 12 spectra recorded over 60 min, are shown in (**D‐F**) and (**J‐L**). [2,3‐^2^H_2_]fumarate was infused at three different concentrations, ranging from 0.1 (n=3) (**A,D,G,J**), 0.3 (n=3) (**B,E,H,K**) and 0.5 g/kg (n=3) (**C,F,I,L**), and started 5 min following the start of acquisition of the first spectrum.
**Figure S2.** Representative ^2^H spectra acquired after injection of 0.3 g/kg fumarate and 24 h after treatment with 0.8 mg/kg MEDI3039. A single spectrum, the sum of 2 spectra and the sum of 4 spectra, recorded over 5 min (**A**), 10 min (**B**) and 20 min (**C**) respectively, are shown, starting at 20 min following fumarate administration. The labeled fumarate concentrations at these times were 2.3, 2.6 and 2.7 mM and the labeled malate concentrations 1.0, 1.0 and 1.1 mM respectively.
**Figure S3.** Tumor malate concentrations before and after treatment with 0.1, 0.4, and 0.8 mg/kg MEDI3039 between 20 and 60 minutes after injecting increasing fumarate concentrations (0.1, 0.3 and 0.5 g/kg) (n=3 per group). The malate concentration is the dependent variable and was assessed at increasing concentrations of fumarate at each of the MEDI3039 drug concentrations. Data are presented as mean ± SD, *P < 0.05, ***P < 0.001, ****P < 0.0001. ns, not significant.
**Figure S4.** Malate/fumarate signal ratios before (untreated n=3) and after MEDI3039 treatment at (**A**) 0.1 (n=3), (**B**) 0.4 (n=3, *P < 0.026, ****P < 0.0001) and (**C**) 0.8 mg/kg (n=3, ****P < 0.0001). Ratios were obtained by summing the fumarate and malate signals between 20 and 60 min after injection of [2,3‐^2^H_2_]fumarate at 0.1, 0.3 and 0.5 g/kg. The malate/fumarate ratio is the dependent variable and was assessed at increasing concentrations of fumarate at each of the MEDI3039 drug concentrations. Data are presented as mean ± SD. ns, not significant. The same untreated controls are shown in the three panels (**A** – **C**).
**Figure S5.** Representative time course data for labeled fumarate, malate and water concentrations with the corresponding coefficients of variance, following injection of increasing concentrations of [2,3‐^2^H_2_]fumarate (**A ‐ C**) 24 h after treatment with 0.8 mg/kg of MEDI3039. The data for a single animal is shown for each fumarate concentration. The coefficients of variance were generated by the AMARES toolbox.

## References

[1] Wheeler JA , Stephens LC , Tornos C , et al. ASTRO Research Fellowship: apoptosis as a predictor of tumor response to radiation in stage IB cervical carcinoma. Int J Radiat Oncol Biol Phys. 1995;32(5):1487‐1493. doi:10.1016/0360-3016(95)00156-s 7635794

[2] Dowsett M , Archer C , Assersohn L , et al. Clinical studies of apoptosis and proliferation in breast cancer. Endocr Relat Cancer. 1999;6(1):25‐28. doi:10.1677/erc.0.0060025 10732783

[3] Gerwing M , Herrmann K , Helfen A , et al. The beginning of the end for conventional RECIST—novel therapies require novel imaging approaches. Nat Rev Clin Oncol. 2019;16(7):442‐458. doi:10.1038/s41571-019-0169-5 30718844

[4] Curran SD , Muellner AU , Schwartz LH . Imaging response assessment in oncology. Cancer Imaging. 2006;6(Special Issue A):S126‐S130. doi:10.1102/1470-7330.2006.9039 17114065 PMC1805076

[5] Zhang D , Jin Q , Jiang C , Gao M , Ni Y , Zhang J . Imaging cell death: focus on early evaluation of tumor response to therapy. Bioconjug Chem. 2020;31(4):1025‐1051. doi:10.1021/acs.bioconjchem.0c00119 32150392

[6] Gallagher FA , Kettunen MI , Hu D‐E , et al. Production of hyperpolarized [1,4‐^13^C_2_]malate from [1,4‐^13^C_2_]fumarate is a marker of cell necrosis and treatment response in tumors. Proc Natl Acad Sci U S A. 2009;106(47):19801‐19806. doi:10.1073/pnas.0911447106 19903889 PMC2785247

[7] Witney TH , Kettunen MI , Hu DE , et al. Detecting treatment response in a model of human breast adenocarcinoma using hyperpolarised [1‐^13^C]pyruvate and [1,4‐^13^C_2_]fumarate. Br J Cancer. 2010;103(9):1400‐1406. doi:10.1038/sj.bjc.6605945 20924379 PMC2990617

[8] Hesse F , Somai V , Kreis F , Bulat F , Wright AJ , Brindle KM . Monitoring tumor cell death in murine tumor models using deuterium magnetic resonance spectroscopy and spectroscopic imaging. Proc Natl Acad Sci U S A. 2021;118(12):e2014631118. doi:10.1073/pnas.2014631118 33727417 PMC8000230

[9] Hesse F , Wright AJ , Somai V , Bulat F , Kreis F , Brindle KM . Imaging glioblastoma response to radiotherapy using 2H magnetic resonance spectroscopy measurements of fumarate metabolism. Cancer Res. 2022;82(19):3622‐3633. doi:10.1158/0008-5472.Can-22-0101 35972377 PMC9530651

[10] Greer YE , Gilbert SF , Gril B , et al. MEDI3039, a novel highly potent tumor necrosis factor (TNF)‐related apoptosis‐inducing ligand (TRAIL) receptor 2 agonist, causes regression of orthotopic tumors and inhibits outgrowth of metastatic triple‐negative breast cancer. Breast Cancer Res. 2019;21(1):27. doi:10.1186/s13058-019-1116-1 30777098 PMC6380056

[11] Hesse F , Wright AJ , Bulat F , Somai V , Kreis F , Brindle KM . Deuterium MRSI of tumor cell death in vivo following oral delivery of (2) H‐labeled fumarate. Magn Reson Med. 2022;88(5):2014‐2020. doi:10.1002/mrm.29379 35816502 PMC9545469

[12] Garwood M , DelaBarre L . The return of the frequency sweep: designing adiabatic pulses for contemporary NMR. J Magn Reson. 2001;153(2):155‐177. doi:10.1006/jmre.2001.2340 11740891

[13] Vanhamme L , van den Boogaart A , Van Huffel S . Improved method for accurate and efficient quantification of MRS data with use of prior knowledge. J Magn Reson. 1997;129(1):35‐43. doi:10.1006/jmre.1997.1244 9405214

[14] Purvis LAB , Clarke WT , Biasiolli L , Valkovič L , Robson MD , Rodgers CT . OXSA: an open‐source magnetic resonance spectroscopy analysis toolbox in MATLAB. PLoS ONE. 2017;12(9):e0185356. doi:10.1371/journal.pone.0185356 28938003 PMC5609763

[15] Lu M , Zhu XH , Zhang Y , Mateescu G , Chen W . Quantitative assessment of brain glucose metabolic rates using in vivo deuterium magnetic resonance spectroscopy. J Cereb Blood Flow Metab. 2017;37:3518‐3530. doi:10.1177/0271678x17706444 28503999 PMC5669347

[16] Teipel JW , Hass GM , Hill RL . The substrate specificity of fumarase. J Biol Chem. 1968;243(21):5684‐5694. doi:10.1016/S0021-9258(18)91921-6 5748979

[17] Ajalla Aleixo MA , Rangel VL , Rustiguel JK , de Pádua RAP , Nonato MC . Structural, biochemical and biophysical characterization of recombinant human fumarate hydratase. FEBS J. 2019;286(10):1925‐1940. doi:10.1111/febs.14782 30761759

[18] Chang J , Ormerod M , Powles TJ , Allred DC , Ashley SE , Dowsett M . Apoptosis and proliferation as predictors of chemotherapy response in patients with breast carcinoma. Cancer. 2000;89(11):2145‐2152. doi:10.1002/1097-0142(20001201)89:11<2145::AID-CNCR1>3.0.CO;2-S 11147583

[19] Mohsin SK , Weiss HL , Gutierrez MC , et al. Neoadjuvant trastuzumab induces apoptosis in primary breast cancers. J Clin Oncol. 2005;23(11):2460‐2468. doi:10.1200/jco.2005.00.661 15710948

[20] De Feyter HM , Behar KL , Corbin ZA , et al. Deuterium metabolic imaging (DMI) for MRI‐based 3D mapping of metabolism in vivo. Sci Adv. 2018;4(8):eaat7314. doi:10.1126/sciadv.aat7314 30140744 PMC6105304

[21] Kaggie JD , Khan AS , Matys T , et al. Deuterium metabolic imaging and hyperpolarized ^13^C‐MRI of the normal human brain at clinical field strength reveals differential cerebral metabolism. Neuroimage. 2022;257:119284. doi:10.1016/j.neuroimage.2022.119284 35533826

[22] Bodansky O , Gold H , Zahm W . The toxicity and laxative action of sodium fumarate. J Am Pharm Assoc. 1942;31(1):1‐8. doi:10.1002/jps.3030310101

