# Supplementary material for: Assessment of the sensitivity of 2H MR spectroscopy measurements of [2,3‐2H2]fumarate metabolism for detecting tumor cell death
Source: NMR Biomed. 2023 May 22;36(10):e4965. doi: 10.1002/nbm.4965 (PMC10909471; doi:10.1002/nbm.4965)
Supplement: Supplementary file 1 — Table S1 Coefficients of variance for fumarate Figure S1. 2H MR spectroscopic measurements of labeled fumarate, malate and water concentrations in MDA‐MB‐231 tumors following injection of increasing concentrations of [2,3‐2H2]fumarate (A‐C, G‐I) 24 h after treatment with 0.1 mg/kg (A‐C) (n=3) and 0.4 mg/kg (G‐I) (n=3) MEDI3039. The corresponding representative spectra, the sum of 12 spectra recorded over 60 min, are shown in (D‐F) and (J‐L). [2,3‐2H2]fumarate was infused at three different concentrations, ranging from 0.1 (n=3) (A,D,G,J), 0.3 (n=3) (B,E,H,K) and 0.5 g/kg (n=3) (C,F,I,L), and started 5 min following the start of acquisition of the first spectrum. Figure S2. Representative 2H spectra acquired after injection of 0.3 g/kg fumarate and 24 h after treatment with 0.8 mg/kg MEDI3039. A single spectrum, the sum of 2 spectra and the sum of 4 spectra, recorded over 5 min (A), 10 min (B) and 20 min (C) respectively, are shown, starting at 20 min following fumarate administration. The labeled fumarate concentrations at these times were 2.3, 2.6 and 2.7 mM and the labeled malate concentrations 1.0, 1.0 and 1.1 mM respectively. Figure S3. Tumor malate concentrations before and after treatment with 0.1, 0.4, and 0.8 mg/kg MEDI3039 between 20 and 60 minutes after injecting increasing fumarate concentrations (0.1, 0.3 and 0.5 g/kg) (n=3 per group). The malate concentration is the dependent variable and was assessed at increasing concentrations of fumarate at each of the MEDI3039 drug concentrations. Data are presented as mean ± SD, *P < 0.05, ***P < 0.001, ****P < 0.0001. ns, not significant. Figure S4. Malate/fumarate signal ratios before (untreated n=3) and after MEDI3039 treatment at (A) 0.1 (n=3), (B) 0.4 (n=3, *P < 0.026, ****P < 0.0001) and (C) 0.8 mg/kg (n=3, ****P < 0.0001). Ratios were obtained by summing the fumarate and malate signals between 20 and 60 min after injection of [2,3‐2H2]fumarate at 0.1, 0.3 and 0.5 g/kg. The malate/fumarate ratio is the depen [file NBM-36-e4965-s001.docx]

**Supplementary Information**

**Assessment of the sensitivity of ^2^H MR spectroscopy measurements of [2,3-^2^H_2_]fumarate metabolism for detecting tumor cell death**

Friederike Hesse^1,2^, Alan Wright^†^, Flaviu Bulat^1,3^, Felix Kreis^1^ and Kevin Brindle^1,4*^

**Table S1**

Coefficients of variance for fumarate

| Drug concentration  (mg/kg) | Injected [2,3-^2^H_2_]fumarate concentration (g/kg) | | | |
| --- | --- | --- | --- | --- |
|  | 0.1 | 0.3 | 0.5 | 1 |
| Untreated | 12-21% | 13-19% | 8-16% | 11-19% |
| 0.1 | 16-18% | 15-20% | 8-18% |  |
| 0.4 | 14-21% | 6-20% | 7-18% |  |
| 0.8 | 12-20% | 8-21% | 9-11% | 3-13% |

Coefficients of variance for malate

| Drug concentration  (mg/kg) | Injected [2,3-^2^H_2_]fumarate concentration (g/kg) | | | |
| --- | --- | --- | --- | --- |
|  | 0.1 | 0.3 | 0.5 | 1 |
| Untreated | 27-34% | 27-33% | 13-29% | 11-27% |
| 0.1 | 20-35% | 16-30% | 16-26% |  |
| 0.4 | 14-28% | 10-29% | 14-18% |  |
| 0.8 | 18-27% | 14-31% | 6-18% | 8-12% |

The coefficient of variance represents the ratio of the standard deviation to the mean.

**Supplementary figures**

**
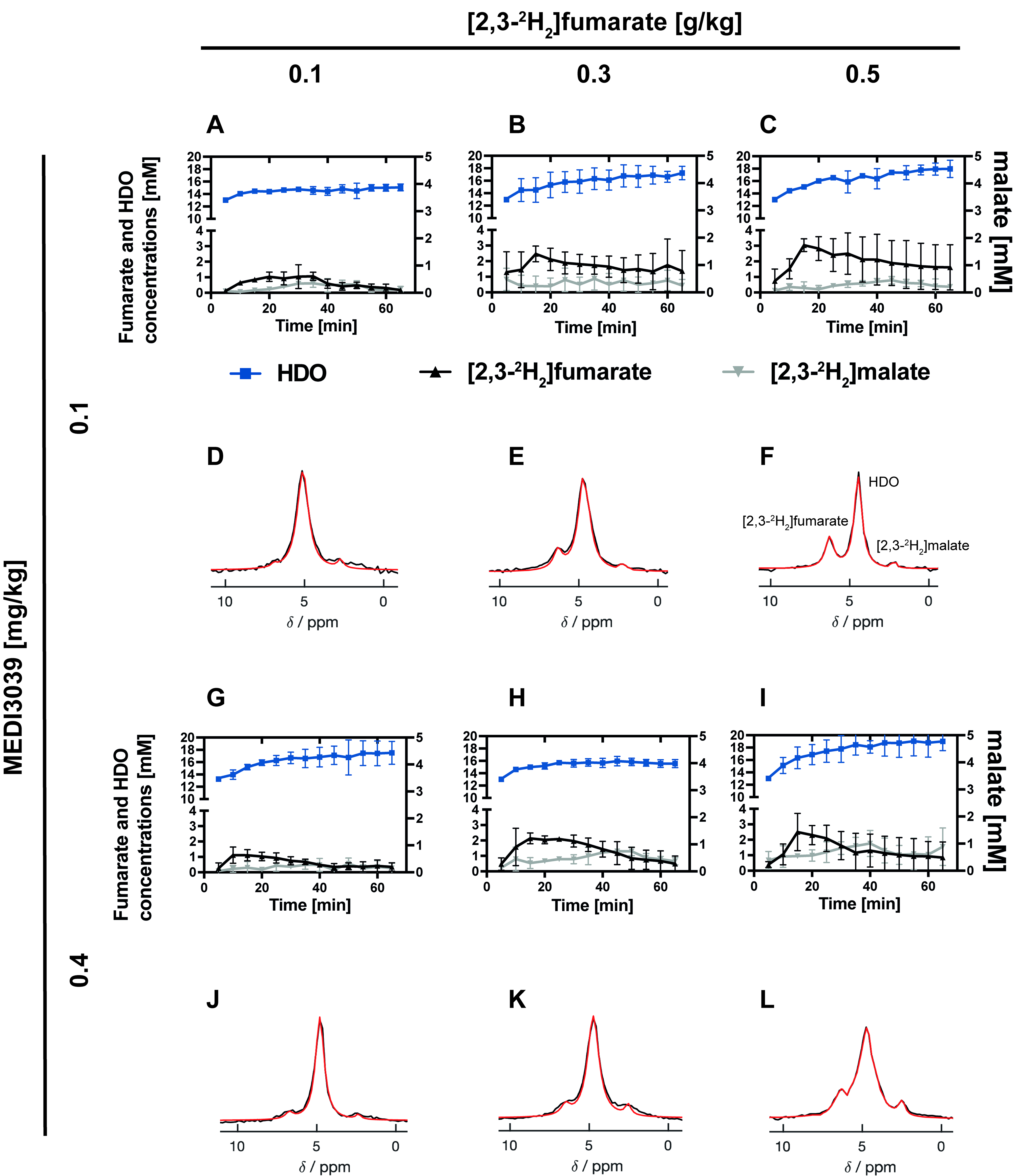
**

**Figure S1.** ^2^H MR spectroscopic measurements of labeled fumarate, malate and water concentrations in MDA-MB-231 tumors following injection of increasing concentrations of [2,3-^2^H_2_]fumarate (**A-C, G-I**) 24 h after treatment with 0.1 mg/kg (**A-C**) (n=3) and 0.4 mg/kg (**G-I**) (n=3) MEDI3039**.** The corresponding representative spectra, the sum of 12 spectra recorded over 60 min, are shown in (**D-F**) and (**J-L**). [2,3-^2^H_2_]fumarate was infused at three different concentrations, ranging from 0.1 (n=3) (**A,D,G,J**), 0.3 (n=3) (**B,E,H,K**) and 0.5 g/kg (n=3) (**C,F,I,L**), and started 5 min following the start of acquisition of the first spectrum.


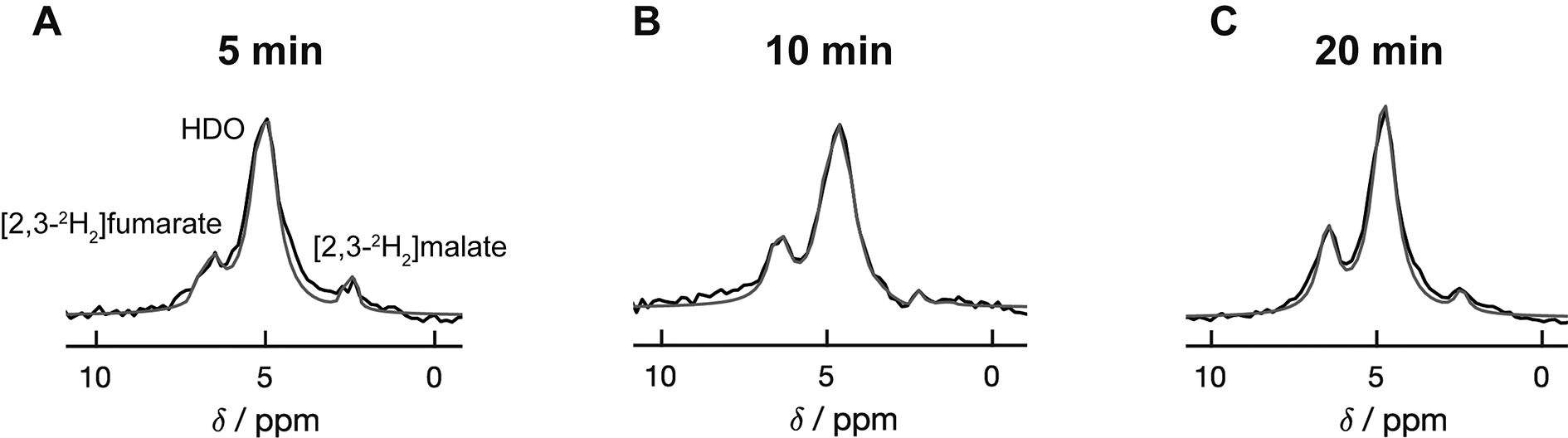


**Figure S2.** Representative ^2^H spectra acquired after injection of 0.3 g/kg fumarate and 24 h after treatment with 0.8 mg/kg MEDI3039. A single spectrum, the sum of 2 spectra and the sum of 4 spectra, recorded over 5 min (**A**), 10 min (**B**) and 20 min (**C**) respectively, are shown, starting at 20 min following fumarate administration. The labeled fumarate concentrations at these times were 2.3, 2.6 and 2.7 mM and the labeled malate concentrations 1.0, 1.0 and 1.1 mM respectively.


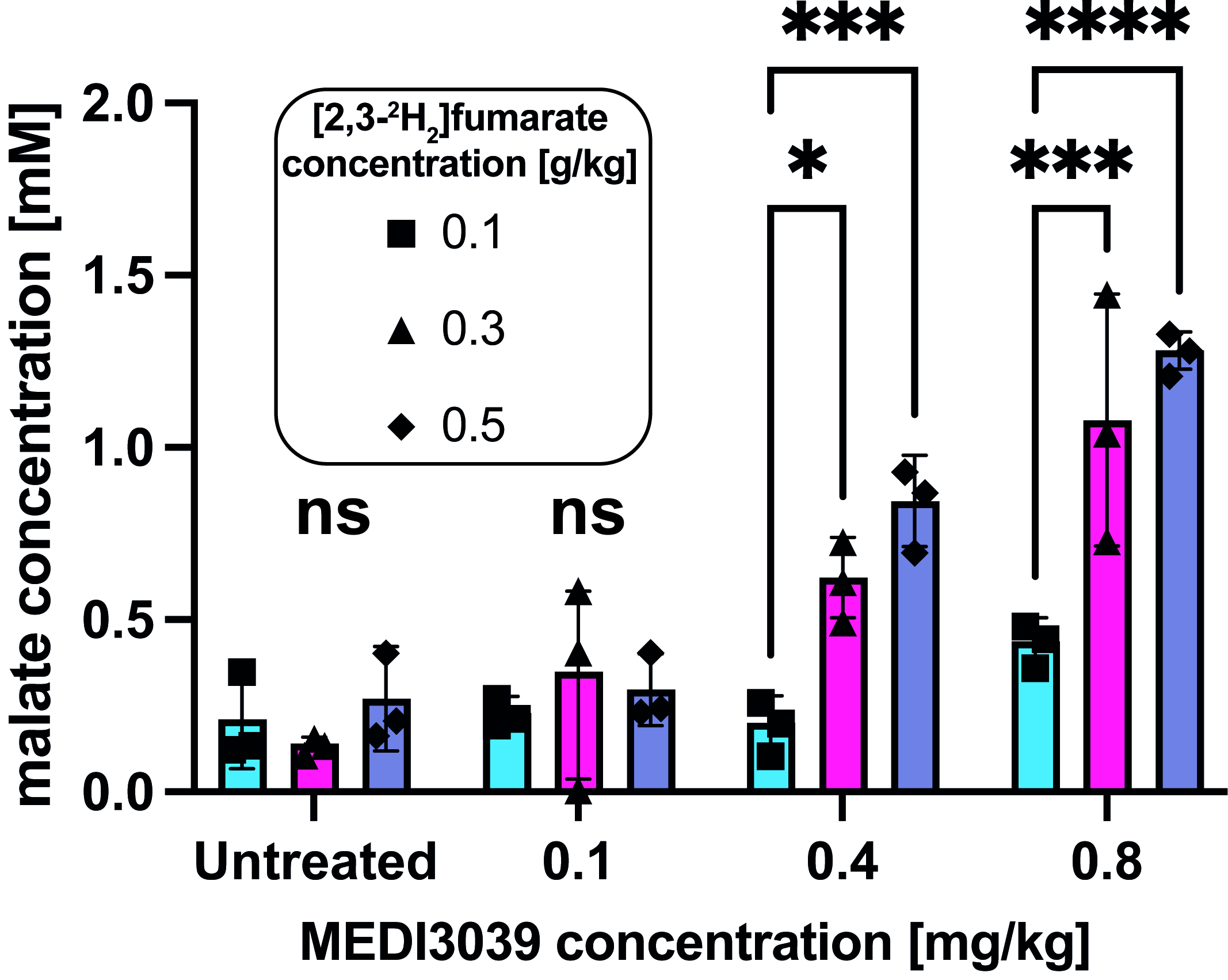


**Figure S3.** Tumor malate concentrations before and after treatment with 0.1, 0.4, and 0.8 mg/kg MEDI3039 between 20 and 60 minutes after injecting increasing fumarate concentrations (0.1, 0.3 and 0.5 g/kg) (n=3 per group). The malate concentration is the dependent variable and was assessed at increasing concentrations of fumarate at each of the MEDI3039 drug concentrations. Data are presented as mean ± SD, *P < 0.05, ***P < 0.001, ****P < 0.0001. ns, not significant.


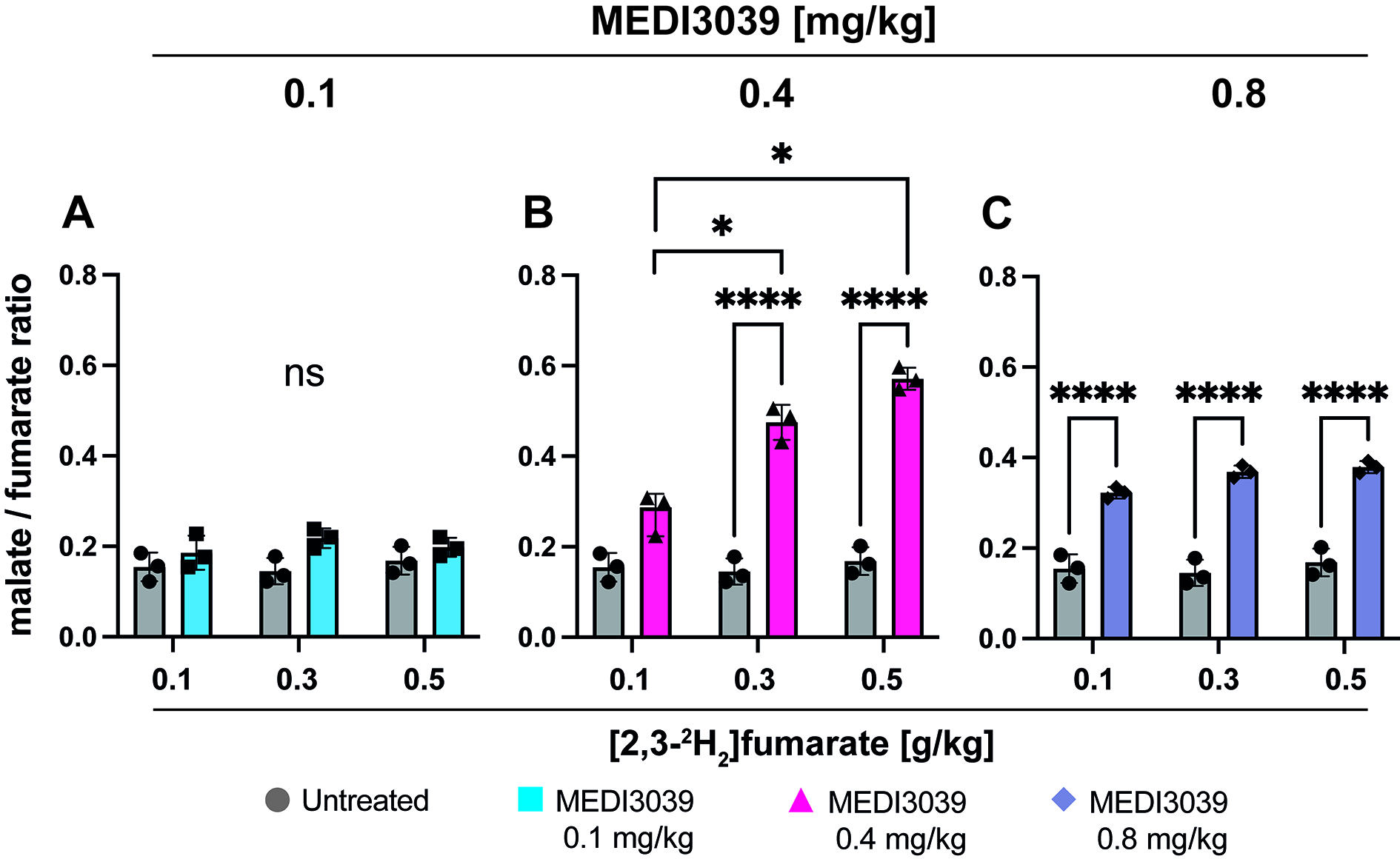


**Figure S4.** Malate/fumarate signal ratios before (untreated n=3) and after MEDI3039 treatment at (**A**) 0.1 (n=3), (**B**) 0.4 (n=3, *P < 0.026, ****P < 0.0001) and (**C**) 0.8 mg/kg (n=3, ****P < 0.0001). Ratios were obtained by summing the fumarate and malate signals between 20 and 60 min after injection of [2,3-^2^H_2_]fumarate at 0.1, 0.3 and 0.5 g/kg. The malate/fumarate ratio is the dependent variable and was assessed at increasing concentrations of fumarate at each of the MEDI3039 drug concentrations. Data are presented as mean ± SD. ns, not significant. The same untreated controls are shown in the three panels (**A** – **C**).


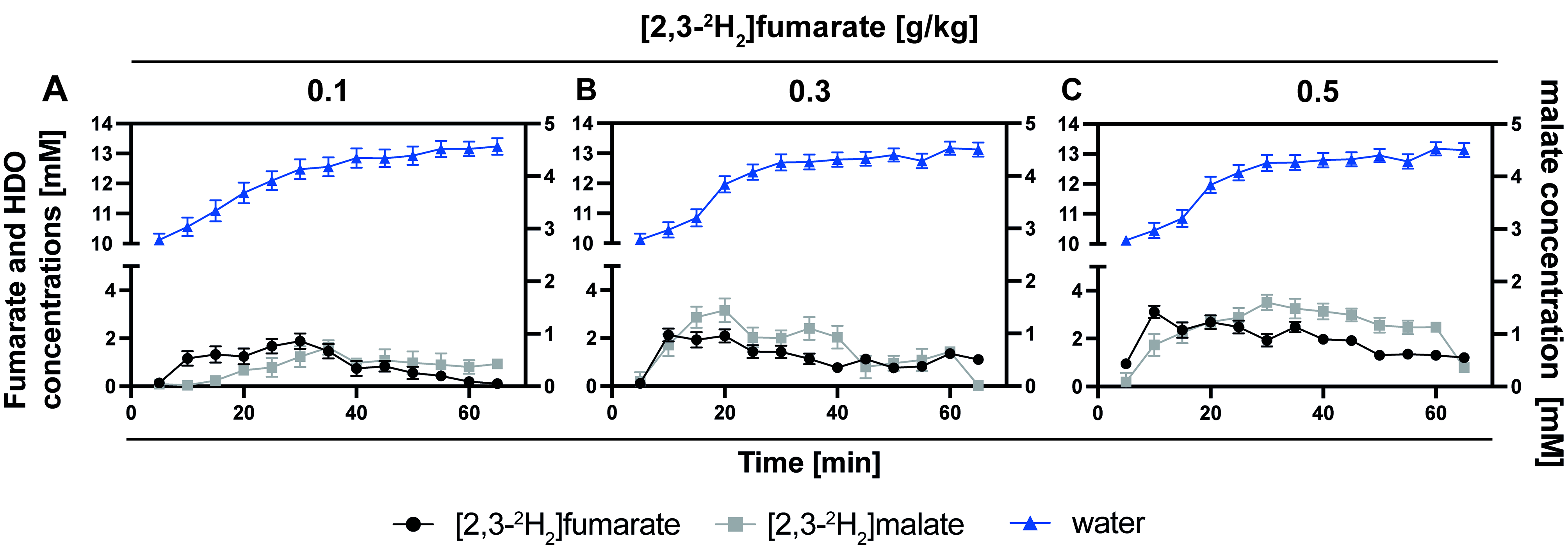


**Figure S5.** Representative time course data for labeled fumarate, malate and water concentrations with the corresponding coefficients of variance, following injection of increasing concentrations of [2,3-^2^H_2_]fumarate (**A - C**) 24 h after treatment with 0.8 mg/kg of MEDI3039. The data for a single animal is shown for each fumarate concentration. The coefficients of variance were generated by the AMARES toolbox.
